# A bifunctional bortezomib-loaded porous nano-hydroxyapatite/alginate scaffold for simultaneous tumor inhibition and bone regeneration

**DOI:** 10.1186/s12951-023-01940-0

**Published:** 2023-06-01

**Authors:** Jiafei Chen, Junru Wen, Yike Fu, Xiang Li, Jie Huang, Xiaoxu Guan, Yi Zhou

**Affiliations:** 1 The Affiliated Hospital of Stomatology, School of Stomatology, Zhejiang University of Medicine, and Key Laboratory of Oral Biomedical Research of Zhejiang Province, Hangzhou, 310006 Zhejiang China; 2 State Key Laboratory of Silicon Materials, School of Materials Science and Engineering, Zhejiang University, Hangzhou, 310027 P.R. China; 3 ZJU-Hangzhou Global Scientific and Technological Innovation Center, Zhejiang University, Hangzhou, 311200 P.R. China; 4 Department of Mechanical Engineering, University College London, London, WC1E 7JE UK

**Keywords:** BTZ/nHA@SA scaffold, nHA, BTZ, Anti-tumor, Bone regeneration

## Abstract

Treatments of osteolytic lesions due to malignant metastasis remain one of the major clinical challenges. The residual tumor cells after surgical resections and an acidic tumor microenvironment are unfavorable for osteogenic induction. Bortezomib (BTZ), a proteasome inhibitor used in chemotherapy, also has an osteogenic potential in concentration- and Ca^2+^-dependent manners. In this study, controlled delivery of BTZ in a novel bifunctional scaffold based on nano-hydroxyapatite (nHA) and sodium alginate (SA) nanocomposite, namely BTZ/nHA@SA, has been explored. By smartly adjusting microenvironments, a sustainable release of Ca^2+^ from nHA could be achieved, which was not only able to cross-link SA but also to regulate the switch between the dual functions of tumor inhibition and bone regeneration of BTZ to promote the osteogenic pathway. The freeze-dried BTZ/nHA@SA scaffold has excellent interconnectivity, is capable to promote the attachment and proliferation of mouse embryonic osteoblast precursor cells, as well as effectively induces breast cancer cell death in vitro. Furthermore, in vivo, studies using a mouse tumor model and a rabbit femoral defect model showed that the BTZ/nHA@SA scaffold could promote tumor ablation, and also enhance bone repair. Therefore, the BTZ/nHA@SA scaffold has unique dual functions of inhibiting tumor recurrence and promoting bone tissue regeneration simultaneously. This smart bi-functional scaffold offers a promising novel approach for oncological treatments by synchronously orchestrating tumor inhibition and tissue regeneration for the repair of neoplastic bone defects.

**Supplementary Information:**

The online version contains supplementary material available at 10.1186/s12951-023-01940-0.

## Introduction

Treatments of osteolytic lesions resulting from malignant metastasis that originate in situ or from a distant organ remain one of the major clinical challenges [1, 2]. The occurrence rate of bone metastasis, such as from breast cancer, gradually increases with postsurgical survival time [3–5]. Bone metastases present a characteristic osteolytic property, tumorous bone defects occur in both primary and metastatic bone tumors inevitably [6, 7]. So extended resection during the traditional surgical performance is an absolute necessity to reduce the occurrence rate [8, 9]. Nevertheless, in situ residual tumor cells can lead to cancer recurrence, and extended bone defects are more susceptible to secondary colonization from tumor metastasis cells. The postoperative recurrence rates are still high [10–12], so chemo/radiotherapy is essential post-surgery [13], although there are several severe drawbacks, including multidrug toxicity/resistance, and ineffective elimination of tumor cells existing in chemo/radiotherapy [14, 15]. Therefore, the treatment of malignant bone tumors remains a huge challenge.

A safe and effective method for cancer treatment is minimally invasive, achieving both tumor killing and bone repairing synchronously to overcome the time mismatch in tumor inhibition and bone defect reconstruction. The emergence of nanomedicine has brought a new approach to cancer therapies [16, 17]. The nanomaterials not only have permeability to kill residual or recolonized tumor cells but also change the undesired tumor microenvironment (i.e. acidic pH) for favoring osteogenesis and bone reconstruction [18–20].

A range of nanomaterials has been invented for novel therapeutic approaches, such as photodynamic therapy (PDT), photothermal therapy (PTT), and chemodynamic therapy (CDT) [21, 22]. MoS_2_ nanoplates acted as photothermal agents in a bi-functional MS-AKT scaffold, owing capability to kill residual tumors as well as to promote osteoblast adhesion, proliferation, and differentiation for accelerated bone tissue regeneration [23]. However, most photothermal agents have limitations, such as rapid degradations, potential toxic degradation products, low efficiency of photothermal conversion, and high temperature generated [24, 25]. These shortcomings could cause severe cellular damage and hinder osteogenesis. A better treatment should be able to inhibit tumor cells and stimulate neoplastic bone defect regeneration simultaneously.

Bortezomib (BTZ), also known as Velcade^®^, is a proteasome inhibitor used in chemotherapy and has been approved by the FDA. It has dual functions of excellent anti-tumor effects [26] and osteogenic activity [27]. The anti-tumor effect of BTZ is based on the inhibition of proteasome activity by blocking NF-κB and JAK–STAT signaling pathways, which results in cell cycle arrest and apoptosis [28, 29]. Clinical studies revealed the potential of BTZ in promoting bone metabolic activity. Oyajobi et al. [30] first reported that BTZ can stimulate new bone regeneration in newborn mouse skulls. Terpos et al. found that the serum ALP and OC levels increased while DKK1 decreased in 34 patients with relapsed multiple myeloma, suggesting varied biological effects of BTZ on different cells [31], thus can be further explored as a novel therapeutic strategy for neoplastic bone defects repair. The challenge is, as a protease inhibitor, BTZ can inhibit Caveolin-1 (CAV1), an essential protein to regulate intracellular calcium homeostasis [32, 33]. CAV1 inhibition can lead to a decreased intracellular Ca^2+^ concentration, which is counterproductive to osteogenesis [34]. This side effect of BTZ needs to be overcome in designing a suitable drug carrier, such as to compensate for the Ca^2+^ loss by supplying Ca^2+^ in the treatment therapy.

In addition to supplying Ca^2+^, reversing an acidic microenvironment from tumors into an osteogenic (alkaline) microenvironment is another huge challenge. The tumor microenvironment is weakly acidic due to the immune inflammatory response and high glycolysis around the tumor, while a favorable osteogenic microenvironment requires weak alkalinity [35]. Therefore, the ability to regulate the pH is key to reversing osteolysis to predominant osteogenesis. Nano-hydroxyapatite (nHA) is the main inorganic component of human bone [36], and dynamically participates in calcium and phosphorus metabolism to mediate bone resorption and reconstruction [37, 38]. nHA can act as a mineral reservoir for releasing alkali ions (Ca^2+^), making it an ideal material for neutralizing the acidic tumor microenvironment and promoting bone formation.

Sodium alginate (SA) is a natural polysaccharide with excellent biocompatibility and biodegradability [39, 40], and has a wide range of applications in a variety of fields from drug delivery to tissue engineering [41]. SA is negatively charged and highly hydrophilic and can be cross-linked rapidly in the presence of divalent cations such as Ca^2+^ to form a gel with stress-relaxation properties [42]. By closely resembling an extracellular matrix, SA is an ideal carrier for cells [43], but has low mechanical strength and lacks tissue adhesion sites [44, 45]. By reinforcing with mineral nanoparticles (i.e. nHA) in SA, the produced composite can not only improve mechanical properties but also promote osteogenic mineralization.

**Fig. 1:**
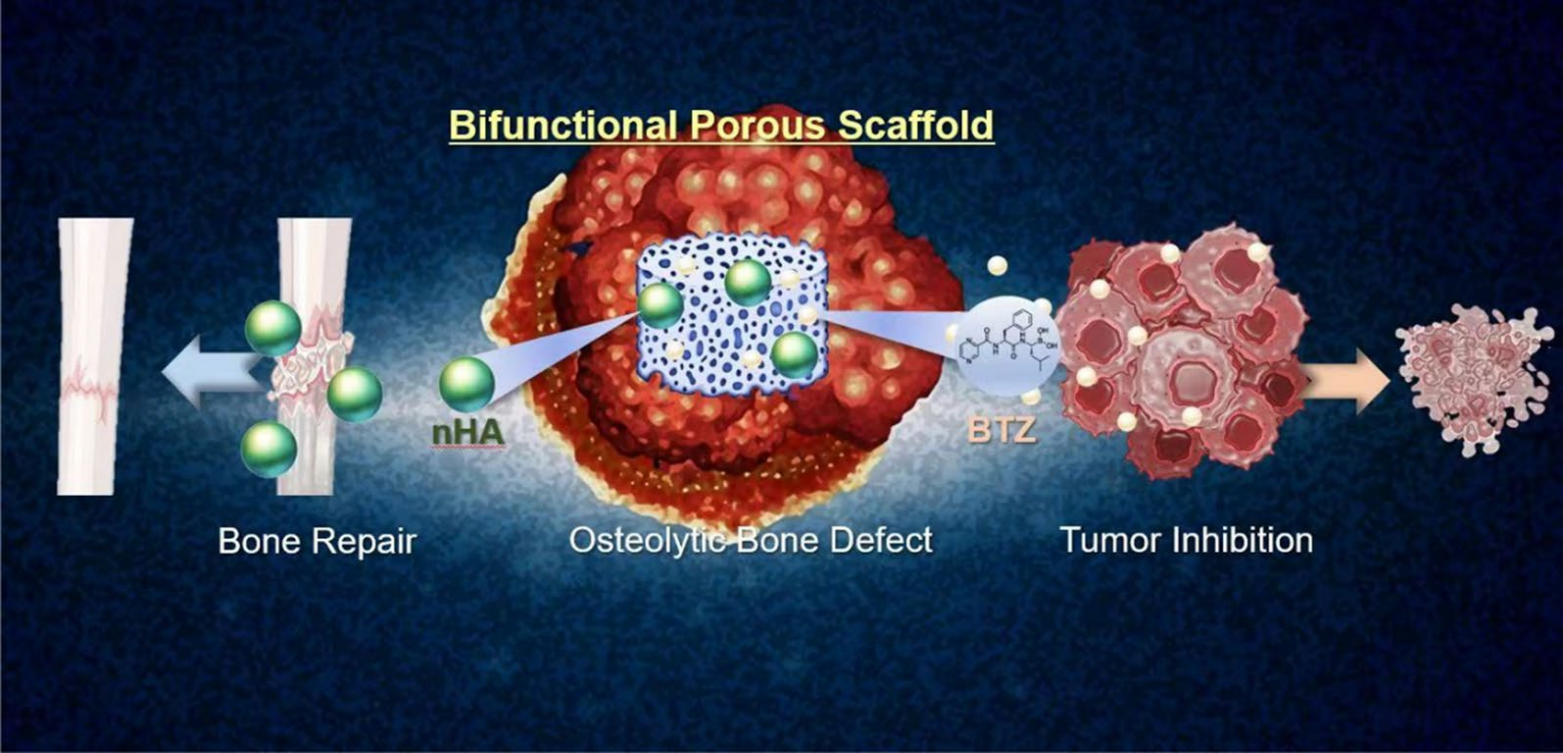
Schematic illustration of BTZ/nHA@SA scaffold for simultaneous tumor inhibition with bone regeneration

Thus, we developed a porous nHA@SA composite scaffold for the smart delivery of BTZ. Glucono-δ-lactone (GDL), was introduced in the nHA@SA system to achieve homogeneous gelation and controlled delivery as it hydrolyses into gluconic acid at a rate dependent on pH [46]. nHA nanoparticles were uniformly dispersed in SA; mediated by GDL, Ca^2+^ ions were slowly and partially released from nHA and cross-linked with the alginate hydrogel. After incorporating BTZ and freeze-drying, a highly porous BTZ/nHA@SA scaffold was obtained with a uniform microstructure and good handling properties (Fig. 1). The BTZ/nHA@SA composite scaffold showed a favorable tumor-killing effect and excellent osteogenic performance from in vivo studies using a mouse tumor model and a rabbit bone defect mode. Compared with the monotherapy via BTZ delivery or nHA@SA scaffold, the drug-loaded composite scaffold presented a higher osteogenic activity than expected. All in all, these results suggest that the BTZ/nHA@SA composite scaffold offers a promising therapeutic approach for neoplastic bone defects.

## Experimental section

### Chemical and reagents

Ethanol and sodium alginate were purchased from Aladdin Reagents (China). Gluconolactone was purchased from Chembee (China). Bortezomib was purchased from D&amp (China). Nano-hydroxyapatite was purchased from RHAWN (China). Trizol, Radio immunoprecipitation assay (RIPA) lysis buffer, BCIP/NBT alkaline phosphatase chromogenic Kit, Alizarin Red S Staining Kit, Calcium Colorimetric Assay Kit, and Dexamethasone (≥ 99%, Reagent grade) were purchased from Beyotime Biotechnology (China). Sodium β-glycerophosphate was purchased from Macklin (China). l-ascorbic acid was purchased from Sigma (China). Calcein AM & propidium iodide (PI) probes were purchased from Life Technologies (China). Reverse Transcription Kit, SYBR Green Detection System Kit, and ECL Chemiluminescent Substrate Kit were purchased from Servicebio (China). PVDF was purchased from Millipore (USA).

### Synthesis of nHA@SA system and BTZ/nHA@SA scaffolds

For the preparation of nHA@SA hybrid system, the 2.5% (w/v) SA was dissolved in distilled water (DW) and stirred at 1000 rpm at room temperature. nHA powder at a designed ratio (W_nHA_/W_SA_ = 1) based on a previous study [47] was gradually added to the SA solution, stirred and mixed continuously until a homogenous suspension was obtained. Then, nHA@SA hydrogel composite samples were prepared by casting the composite suspension into discs, cylinders, and films for in vitro and in vivo studies, which were sterilized using an ultraviolet (UV) irradiation (253.7 nm) for 1 h before testing.

To prepare the BTZ/nHA@SA scaffold, BTZ was dissolved in DMSO first to make a 1 M stock solution. To load the drug, the calculated amounts of BTZ (0.4, 0.8, 0.12, 0.2, and 0.24 μg) were added into 100 mg nHA@SA hydrogel mixture, stirred at 200 rpm for 24 h at room temperature. Then 0.5% GDL was added, further stirred at 37 °C and 500 rpm for 5 min to ensure a slow and gradual release of Ca^2+^ from nHA, thus leading to an overall homogeneous cross-linking of SA. Finally, the resultant mixture was transferred into a 24-well plate and left in quiescence at room temperature for 6 h to form solid gels.

Afterward, hydrogel samples were frozen at − 20 °C for 2 h, followed by − 80 °C for 4 h, and then freeze-dried in a Freeze-dryer (FD-250101, FTFDS) at − 50 °C for 48 h to obtain porous scaffold. The samples were sterilized under UV irradiation for 2 one-hour cycles before the biological testing.

### Structural characterization

The microstructure of BTZ/nHA@SA scaffold was examined using a field-emission scanning electron microscope (FESEM, Apreo 2S, Thermo Scientific, USA). A small piece of freeze-dried scaffold material was painted with a conductive adhesive and sputter coated with gold. The elemental mapping was obtained by Energy-Dispersive Spectrometry with SEM (EDS, QUANTAX, Brucker, GER). The phase identification analysis of freeze-dried scaffold samples was characterized using an X-ray diffractometer (XRD, X’pert PRO MPD, Netherlands), with Cu Kα radiation (λ = 0.154 nm) and operating at 40 kV and 40 mA, scanning 2θ from 10° to 80°

### Swelling test

Each nHA@SA freeze-dried scaffold sample was weighed and recorded as W_0_, then placed in a 10 mL of phosphate buffer solution (PBS) at the pH values of 6.5 and 7.4, respectively, in a 15 ml centrifuge tube. The tubes were kept at 37 °C for various time points (1, 2, 3, 6, 12, 24, 36, and 48 h). At each time point, the hydrogel sample was removed from the tube, drained with filter paper, then weighed and recorded as W_t_. The swelling ratio of the scaffolds was calculated using the following equation:

$${\text{Swelling ratio}} = \left( {{\text{W}}_{\text{t}} - {\text{W}}_0 } \right)/{\text{W}}_0 \times {1}00\% .$$

### Degradation test

Each nHA@SA scaffold was weighted and recorded as W_0_. Then the scaffolds were placed into the PBS solutions with pH values of 6.5 and 7.4 for 2 h on a shaking incubator at 37 °C and 100 rpm. The PBS solutions were refreshed every day. At the time point of 2, 4, 6, 8, 10, 12, 14, 16, 20, and 24 days, the scaffold samples were collected, washed with DW (2 times), and then stored in a − 20 °C refrigerator. Finally, the collected scaffolds were freeze-dried, then weighed and recorded as W_t_ accordingly. The degradation rate of nHA@SA scaffold was calculated according to the formula:

$${\text{Weight loss}} = \left( {{\text{W}}_0 - {\text{W}}_{\text{t}} } \right)/{\text{W}}_0 \times {1}00\% .$$

### Mechanical testing

The SA scaffold and nHA@SA scaffold were made in the 10 mm height and 10 mm diameter molds. The compressive tests of the materials were determined by an Electronic Universal Testing Machine (INSTRON 5982, UK), for which the compressive speed was set at 1 mm/min and no preload was applied at room temperature. During the experiment, the stress–strain curve and the highest compressive stress were recorded.

### Ca^2+^ releasing test

To assay the release of Ca^2+^ in vitro, 30 mg nHA@SA scaffolds were dispersed in 2 ml PBS solutions with pH values of 6.5 and 7.4 on a shaking incubator at 37 °C and 100 rpm. At every predetermined time (0, 6, 12, 24, 36, 48, 72, and 96 h), 500 μl supernatant was collected for analysis, and an equal fresh buffer was added to keep the constant volume. Finally, the ionic concentrations of Ca^2+^ in the graded extracts were investigated by Calcium Colorimetric Assay Kit.

### BTZ loading and releasing test

The characteristic absorption peaks of the nHA@SA scaffold and BTZ/nHA@SA scaffold and BTZ standard solutions (0, 5, 10 15, 25, and 30 nM) were analyzed by a UV–Vis spectrophotometer (UV-2600, Shimadzu, Japan). BTZ/nHA@SA scaffold was immersed in DI water on a shaking incubator (37 °C, 100 rpm). Subsequently, 3 ml of supernatant was taken and replaced with 3 ml of fresh DI water. The cumulative release of BTZ released was measured with a UV–Vis spectrophotometer, and the encapsulation rate of BTZ in BTZ/nHA@SA scaffold was calculated. Scaffolds were immersed in PBS solutions of different pH values (6.5 and 7.4). At pre-determined time intervals (2, 4, 6, 8, 10, 12, 14, 24, 36, 48, and 60 h), aliquots (3 ml) were withdrawn and replaced with fresh medium. The absorption peaks at 270.5 nm of the supernatant was measured. The drug release amount at each time point was calculated according to the drug standard curve.

### In vitro cell culture

Mouse breast cancer cells (4T1) and mouse embryonic osteoblast cells (MC3T3) were cultured with RPMI-1640 and DMEM respectively, both were supplemented with 10% fetal bovine serum (FBS) and 1% penicillin and streptomycin.

Osteogenic differentiation induction (ODI) medium composed of α-MEM medium, 10% FBS, 1% penicillin/streptomycin, 10 mM β-glycerophosphate, 50 mg/ml l-ascorbic acid, and 100 nM dexamethasone, was used for the induction of MC3T3 cells osteogenic differentiation.

The extract medium from BTZ/nHA@SA scaffold was obtained by immersing 5 mg scaffolds with various drug loading (0, 0.02, 0.04, 0.06, 0.1, and 0.12 μg) in 10 ml culture medium for 24 h, the drug released levels were correspondent to the concentrations of BTZ standard solution (0, 5, 10, 15, 25, and 30 nM).

### In vitro anti-tumor experiment

4T1 cells (1 × 10^4^ cells per well) were cultured with various concentrations of BTZ (0, 5, 10 15, 25 and 30 nM) and the extract medium from test scaffolds at 37 °C with 5% CO_2_. Cell Counting Kit-8 assay was used to measure the cell viabilities. The absorbance at 450 nm was measured by a microplate reader (Epoch, Biotek).

Live and dead staining assay was also performed after 24 h culture. The 4T1 cells were washed with PBS, and incubated with 100 μl Calcein/PI dilutions at room temperature for 30 min in the dark, then the viability of the cells in each group was observed using an inverted fluorescent microscope (Ts2R-FL, Nikon, Japan).

### In vitro biocompatibility of osteoblast cells

MC3T3 cells (1 × 10^4^ cells per well) were cultured with various concentrations of BTZ and were tested first. MC3T3 cells were cultured in the scaffold extracts medium at 37 °C with 5% CO_2_ for 24 h. CCK-8 and live-dead staining assays (as described in “BTZ loading and releasing test” section) were performed.

To further evaluate the biocompatibility of BTZ/nHA@SA scaffold, MC3T3 cells were seeded directly on the scaffolds and cultured for 1, 2, and 3 days. The viability of cells on the scaffold was determined by live-dead staining with Calcein/PI.

At each time point, MC3T3 cells cultured on BTZ/nHA@SA scaffold were fixed with 2.5% glutaraldehyde in PBS overnight, and post-fixed with 1% Osmium tetroxide (OsO_4_) in PBS for 2.5 h. Subsequently, samples were orderly dehydrated using a graded series of ethanol (30%, 50%, 70%, 80%, 90%, 95%, 100%) for 20 min, and dried with a critical point dryer (HCP-2, Hitachi, Holland). After Au coating, the cell morphology on the scaffold was observed under a FESEM.

### In vitro osteogenic differentiation and mineralization

The effects of BTZ released from the scaffolds on the differentiation and mineralization of MC3T3 cells were investigated using alkaline phosphatase (ALP), Alizarin red staining, and gene expression.

ALP staining was performed with a BCIP/NBT alkaline phosphatase chromogenic Kit. 1 × 10^5^ MC3T3 cells were cultured with the extract medium and ODI medium in 24-well plates for 7 days. Then the cells were fixed with 4% paraformaldehyde (PFA) for 30 min, and stained with ALP staining solution. After washing with distilled water, the ALP-stained cells were photographed.

ARS staining was performed with Alizarin Red S Staining Kit. 1 × 10^5^ MC3T3 cells were cultured with the extract medium in 24-well plates for 14 days. MC3T3 cells were fixed with 4% PFA for 15 min, and stained with Alizarin Red for 20 min at room temperature. After rinsing with distilled water, the images of ARS-stained cells were recorded.

Gene expression of osteogenic differentiation markers, such as ALP and the transcription factor Osterix (*Sp7*) were determined by real-time quantitative Polymerase Chain Reaction (qPCR). 1 × 10^5^ MC3T3 cells were cultured with the extract and ODI medium. After 3, 5, and 7 days of culture, MC3T3 cells were collected in Trizol and the total RNA was extracted. cDNA was generated using a Reverse Transcription Kit. qPCR was performed using the SYBR Green Detection System kit (n = 3). The primers were listed in Additional file 1: Table S1.

In addition, a western blot analysis of RUNX2, COL1, and GAPDH was carried out. 1 × 10^5^ MC3T3 cells in 24-well plates were cultured with the extract medium for 7 days, then the cells were collected and lysed by WB/IP lysis buffer at 4 °C for 10 min. Subsequently, 40 μg protein of each sample was loaded onto the 10% SDS-PAGE at 80 V for 1.5 h, then the proteins were transferred onto the PVDF membrane. After blocking with 5% milk for 2 h, the PVDF membrane was incubated with primary antibodies at 4 °C for 12 h. Primary antibodies against the following proteins were used: RUNX2 (Mouse, 1:750), COL1 (Mouse, 1:750), and GADPH (Mouse, 1:45,000). Then the secondary antibodies (Mouse, 1:3000) were utilized to incubate with the membrane at room temperature for 2 h. The bands were visualized using an ECL Chemiluminescent Substrate Kit, followed by quantification analysis using the Image J software.

### In vivo study

All animal experiments were carried out according to the guidelines for the use and care of laboratory animals approved by the ethics committee of the Biological Resource Centre of the Agency for Science, Technology and Research, Zhejiang University. Female Balb/c mice (4 weeks old) were purchased from Shanghai SLAC Laboratory Animal Co. Ltd. The 8-week-old male rabbits were purchased from the Zhejiang Academy of Medical Sciences animal center. All animals were humanely treated during the experiments.

### In vivo anti-tumor experiment

RPMI-1640 medium with 10% FBS was used to cultivate 4T1 cells. After acclimation for 5 days, 4-week-old mice had their axilla debrided, and the 4T1 tumor cells (2 × 10^6^) were implanted into their axillae. Anti-tumor experiments were started when the tumor volume reached 100 mm^3^.

Mice were divided randomly into five groups (n = 5): (I) Ctrl, sham operation; (II) nHA@SA scaffold implantation; (III) injection of BTZ; (IV) BTZ injection + nHA@SA scaffold implantation; and (V) BTZ/nHA@SA scaffold implantation. The tumor volume and weight of mice were assessed and recorded every two days. The tumor volume (V) was calculated using the following formula: volume = (tumor length) × (tumor width)^2^ × 0.5 [48]. The relative volume of the tumor was calculated as the tumor volume at a specific day over the tumor volume at day 0. Blood from the mouse ocular vein was collected on the 14th day following the standard serum collection procedure.

At the end of the experiments, the tumor and main organs (heart, liver, spleen, lung, and kidney) were collected and treated with a 10% formalin solution. Cancer tissue and viscera were embedded in paraffin first, and 4 μm sections were obtained using an Ultra-Thin Semiautomatic Microtome (RM2016, Leica). The sections were stained with haematoxylin/eosin (H&E) and examined using an inverted fluorescence microscope.

### In vivo osteogenesis experiment

A critical-size femoral defect model [49] was used in the osteogenesis study. The bone defect (6 mm in diameter × 9 mm in depth) was made at the right distal femur of 8 weeks old male rabbit. The rabbits were randomly divided into three groups (n = 3): (a) BTZ only (no implantation); (b) nHA@SA scaffold implantation; (c) BTZ/nHA@SA scaffold implantation. After 12 weeks, the rabbits were sacrificed by an intraperitoneal injection of 10% chloral hydrate. The femur was collected, fixed in 4% paraformaldehyde at 4 °C for 48 h, and then kept in 75% alcohol in a specimen container. The femur samples were analysed by using micro-CT scanning (SCANCO μCT 100, Scanco Medical, Switzerland; source voltage: 70 kVp, power: 200 μA, exposure time: 300 ms, and voxel size: 30 microns) to determine the bone formation in the defect site. The raw scanned data were reconstructed using the scan Evaluation. A 3D analysis of bone formation was carried out in scan Evaluation, such as bone volume/total bone volume (BV/TV), local bone density (BMD), and trabecular number (Tb.N), trabecular thickness (Tb.Th), trabecular separation (Tb.Sp), and cortical bone thickness (Ct.Th). The osteogenic capacity of the implanted scaffolds in the bone defects was compared.

In addition to micro-CT imaging, the defect sections of rabbit femurs were extracted, fixed in 4% paraformaldehyde at 4 °C for 48 h, and dehydrated with a series of gradient alcohols in a dehydrator (Danotello, DIAPATH). The bone samples were dipped in wax, embedded in paraffin, and kept in the − 20 °C freezer (JB-L5). 4 μm slices were sectioned using an Ultra-Thin Semiautomatic Microtome (RM2016, Leica), then stained with H&E, and analysed using an inverted fluorescence microscope.

To further quantify the new bone formation in the defect site, images of bone sections in the defect site were recorded with a positive white light photography microscope (Eclipse Ci-L, Nikon, Japan). The trabecular bone area (mm^2^) within a defined region of interest (ROI) was measured. The proportion of trabecular bone area was calculated according to the formula: the percentage of the trabecular bone area (%) = trabecular bone area/ROI area * 100% (Fig. 1).

### Statistical analysis

All data were expressed as mean values (± standard deviation) and analysed by GraphPad Prism 8.0. The homogeneity of variances and the linearity of the relationship between dependent and independent variables were verified. The significance level was set at 0.05. Comparisons and significance analysis of multiple groups were conducted through a one-way analysis of variance (ANOVA). *p* < 0.05 was considered statistically significant.

## Results

### Synthesis and characteristics of nHA@SA

**Fig. 2:**
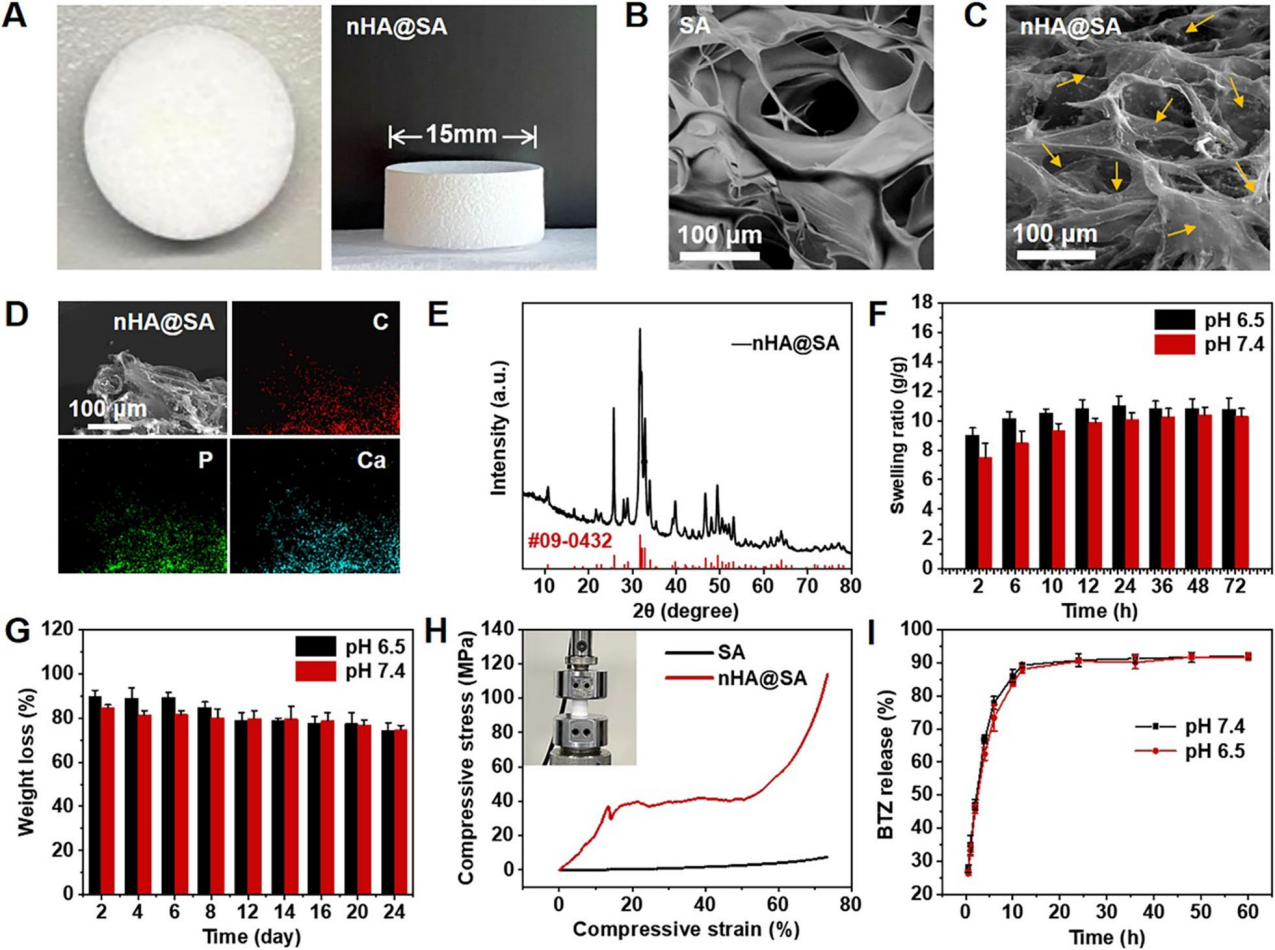
**A** Photographs of nHA@SA scaffold; **B** FESEM images of SA scaffold; **C** FESEM images of nHA@SA scaffold; **D** EDS elemental mapping analysis of Ca and P in nHA@SA scaffold; **E** XRD pattern of nHA and nHA@SA; **F** Swelling ratio of nHA@SA scaffold at pH 6.5 and 7.4; **G** Degradation of nHA@SA scaffold at pH 6.5 and 7.4; **H** Typical compressive stress–strain curves of SA scaffold and nHA@SA scaffold; **I** Drug release curve of BTZ/nHA@SA scaffold at pH 6.5 and 7.4

To ameliorate the delivery of BTZ and improve bone activity simultaneously, we specifically developed a drug-loading material, with SA as the matrix and nHA as the bioactive filler. In preparation of the composite scaffold, GDL was used as the modulator to slowly release Ca^2+^ from the uniformly dispersed nHA particles without introducing other fluid calcium sources, thus ensuring synchronization of cross-linking process and homogeneity of the material. The nHA@SA composite scaffold had a homogeneous interconnected porous structure (Additional file 1: Fig. S1A, Fig. 2A). FESEM showed the internal structure of the scaffold (Additional file 1: Fig. S1B). The scaffold pore size was ranged from 50–200 μm, which allowed osteoblasts to grow into the scaffold. In contrast to the unfilled calcium alginate (Fig. 2B, C), many spherical nanoparticles were uniformly embedded in the struts of the scaffold, indicating that the nanoparticles were successfully loaded, and the rougher surface obtained also provided attachment sites for cells. The EDS element mapping further confirmed that Ca and P were evenly distributed in the scaffold struts. As expected, no P was detected on the SA scaffold (Additional file 1: Fig. S1C), thus demonstrating that the nHA nanoparticles have been successfully incorporated in the nHA@SA composite scaffold (Fig. 2D). This was further confirmed by the XRD analysis of the nHA@SA composites as compared to that of SA only material (Additional file 1: Fig. S1D, Fig. 2E).

### Property evaluations of nHA@SA

Alginate is highly hydrophilic and the degradation rate is pH dependent. The swelling behavior of nHA@SA scaffold was evaluated. At the pH of 6.5, the swelling ratio of nHA@SA scaffold was 900% after 2 h, reached the peak of 1100% after 24 h, and stabilized afterward. In comparison, the swelling ratio at pH 7.4 was lower, 720% after 2 h, and also slower. Both reached the swelling equilibrium after 24 h (Fig. 2F).

The in vitro solubility of the composites was monitored by measuring the weight loss with time (Additional file 1: Fig. S2A, Fig. 2G). The scaffold dissolution rate was lower in a weakly acidic group for up to 12 days. Over time, the speed of scaffold dissolution in both groups become stabilized, especially after 12 days, with no statistical difference between the two groups (*p* > 0.05). The total mass reached 74.7% (pH 7.4) and 74.4% (pH 6.5) of initial weight at 24 days. These results indicated that the relatively low degradation rate of the scaffold may be able to provide continuous structural support for the bone repair and regeneration process and match the osteogenesis process.

The compression test of the scaffold demonstrated that the mechanical strength and elastic modulus of nHA@SA scaffold were significantly higher than those of pure SA scaffold (*p* < 0.05; Fig. 2H, Additional file 1: Fig. S2B, C), the enhancement of which was beneficial to the further regeneration of bone tissue defects.

It is well known that Ca^2+^ supplementation and homeostasis play an important role in the process of bone reconstruction via influencing osteoblast survival and proliferation. As shown in Additional file 1: Fig. S2D, the nHA@SA scaffold continued to release Ca^2+^ at pH 6.5 and 7.4 over time. Ca^2+^ release was relatively fast in an acidic environment (i.e. pH 6.5) due to the accelerated degradation rate of nHA.

The UV absorption BTZ/nHA@SA scaffold was presented in Additional file 1: Fig. S2E and the peak at 270 nm was the characteristic BTZ absorption, indicating that BTZ was successfully loaded. In this study, the encapsulation rate of BTZ was about 77.62% (Additional file 1: Fig. S2F). The result indicated that BTZ/nHA@SA scaffold could efficiently load BTZ. In addition, the drug release kinetics of BTZ/nHA@SA scaffold was simulated in both tumor (i.e. pH 6.5) and osteogenic (i.e. pH 7.4) microenvironments. As can be seen from the drug release curve (Fig. 2I), the BTZ release rate from BTZ/nHA@SA composite reached nearly 90% within 12 h at both pH values, showing the drug release behavior was unaffected by the pH.

### In vitro biocompatibility of BTZ/nHA@SA

**Fig. 3:**
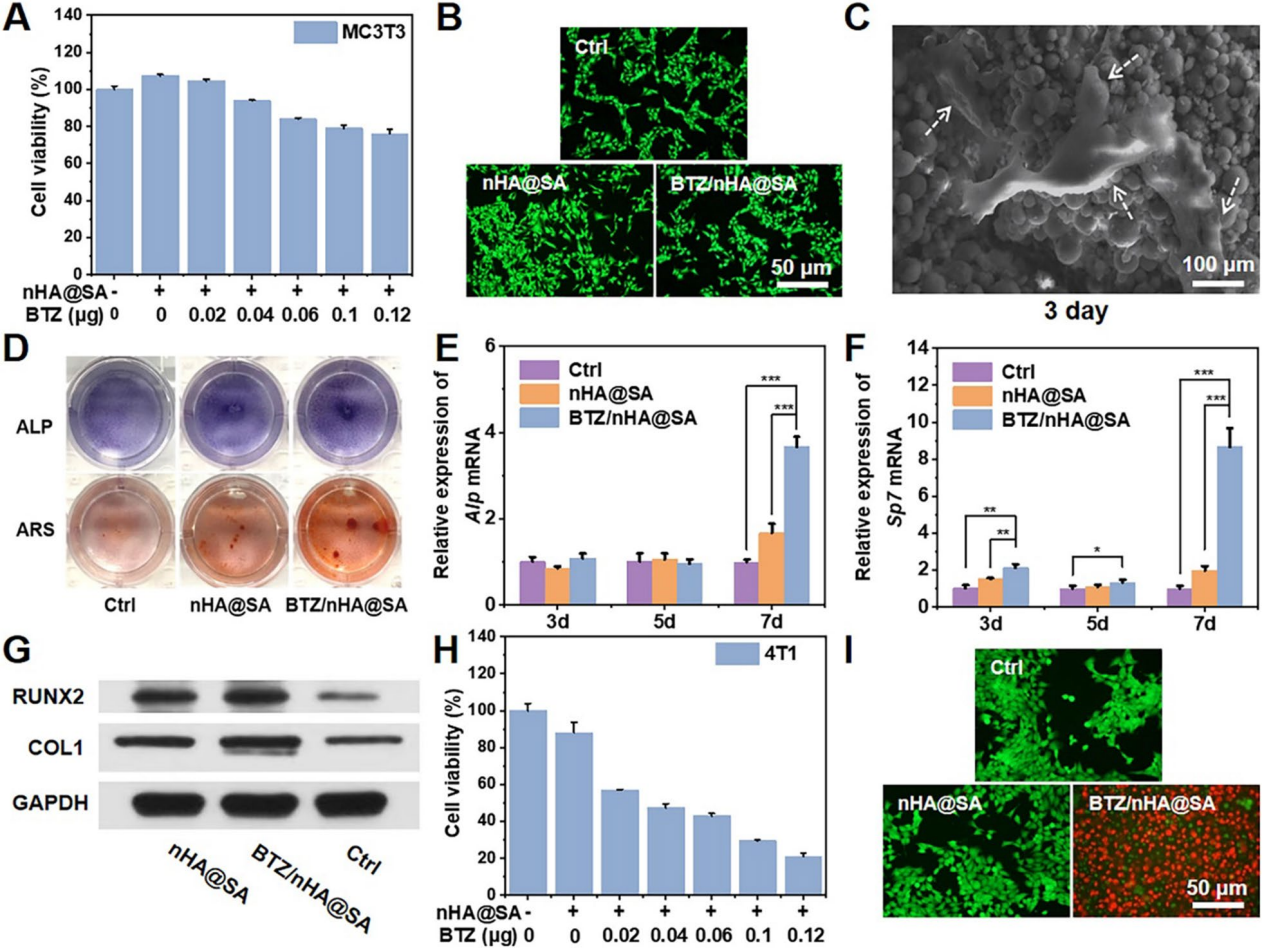
**A** Viabilities of osteoblast cells (MC3T3) cultured with different materials; **B** Fluorescence images of osteoblast cells (MC3T3) stained with Calcein AM (green, live cells) and PI (red, dead cells); **C** SEM images of MC3T3 cells co-cultured with BTZ/nHA@SA scaffold at day 3; **D** Representative images of ALP staining and ARS staining of MC3T3 cells cultured with nHA@SA and BTZ/nHA@SA extract at day 7 or day 14; **E** Relative mRNA expression levels of osteogenic genes *Alp* at day 3, 5 and 7; **F** Relative mRNA expression levels of osteogenic genes *Sp7* at day 3, 5 and 7; **G** Expression levels of RUNX2 and COL1 in MC3T3 cells after 7 days; **H** Viabilities of breast cancer cells (4T1) cultured with different materials; **I** Fluorescence images of breast cancer cells (4T1) stained with Calcein AM (green, live cells) and PI (red, dead cells). *p < 0.05, **p < 0.01, ***p < 0.001

The effect of BTZ released from the BTZ/nHA@SA scaffold was firstly studied. By immersing the scaffold in DMEM for 24 h, the viability of the cells cultured with the extract was measured. Higher viability of MC3T3 cells was found at the low BTZ concentration (5 nM). However, as BTZ concentration increased, the cell viability decreased (Fig. 3A).

The live-dead staining results evaluated by Calcein AM and PI staining assay displayed that MC3T3 cells had vigorous activity in all groups after 24-h culturing with the extract (Fig. 3B).

The MC3T3 cells were then cultured directly on BTZ/nHA@SA scaffold for 1, 2, and 3 days, the Calcein AM/PI staining results were presented in Additional file 1: Fig. S2A. The number of active live cells increased with time, thus confirming the excellent biocompatibility of the scaffold. The attachments of MC3T3 cells on BTZ/nHA@SA scaffold were further examined under SEM with a higher resolution. The rougher surface of the scaffold provided favorable sites for the MC3T3 cells to adhere and spread, as highlighted in Additional file 1: Fig. S3B and Fig. 3C.

The potential of BTZ/nHA@SA on osteogenic activity was conducted. MC3T3 cells were cultured with extract medium from nHA@SA and BTZ/nHA@SA scaffold for 7 and 14 days. Alkaline phosphatase (ALP) activity was higher in BTZ/nHA@SA group after 7 days, and calcium nodule formation capacity was also stronger in BTZ/nHA@SA group after 14 days of culture (Fig. 3D).

Real-time fluorescence quantitative PCR results indicated that the expression of *Alp* and *Sp7* transcription were higher in BTZ/nHA@SA group after 7 days (Fig. 3E, F). The protein expressions of Runt-associated transcription factor 2 (RUNX2) and type I collagen (COL1) were significantly up-regulated in BTZ/nHA@SA group after 7 days (*p* < 0.05; Fig. 3G). Therefore, the nHA@SA scaffold had a positive osteogenic effect, which can be further enhanced by loading a relatively low concentration (10 nM) of BTZ.

### In vitro anti-tumor properties of BTZ/nHA@SA

MC3T3 and 4T1 cells were treated with the BTZ concentrations at 0, 5, 10, 15, 25, and 30 nM, CCK-8 assay revealed that the proliferation rate of 4T1 cells decreased rapidly with the increased BTZ concentrations, while the proliferation rate of MC3T3 cells was proportional to BTZ concentration and reached a peak at 10 nM, and decreased at the concentrations over 15 nM (Additional file 1: Fig. S4).

As displayed in Fig. 3H, the cell activity decreased rapidly while BTZ concentration increased. These results confirmed that the anti-tumor effect of BTZ was maintained in the BTZ/nHA@SA scaffold, while at the BTZ concentration of 5–10 nM in BTZ/nHA@SA scaffold, a synchronous tumors inhibition and enhanced osteogenesis may be obtained.

The live/dead straining results exhibited a strong red fluorescence on the BTZ/nHA@SA scaffold after 24 h culture, displaying a very low cell viability of 4T1 cells in comparison to the high cell activity on the control and nHA@SA scaffold (Fig. 3I), indicating that BTZ/nHA@SA scaffold caused severe damage to tumor cells. These results revealed that both BTZ and BTZ/nHA@SA have cell-specific effects on tumor cells and osteoblasts. For tumor cells, it could work in a concentration-dependent killing way, while for osteoblasts, it can promote cell proliferation at low concentrations (10 nM) but inhibit the proliferation at high concentrations (≥ 15 nM).

Therefore, we speculated that after BTZ/nHA@SA scaffold was implanted in vivo, especially in the first 12 h, a high concentration of BTZ release can kill tumor cells. After an initial burst release, a sustained release of BTZ from the scaffold at low concentrations can mediate osteoblasts proliferation to promote bone repair, thus fulfilling a time-dependent tissue-specific dual bio function of a smart scaffold.

### In vivo anti-tumor effects of BTZ/nHA@SA

**Fig. 4:**
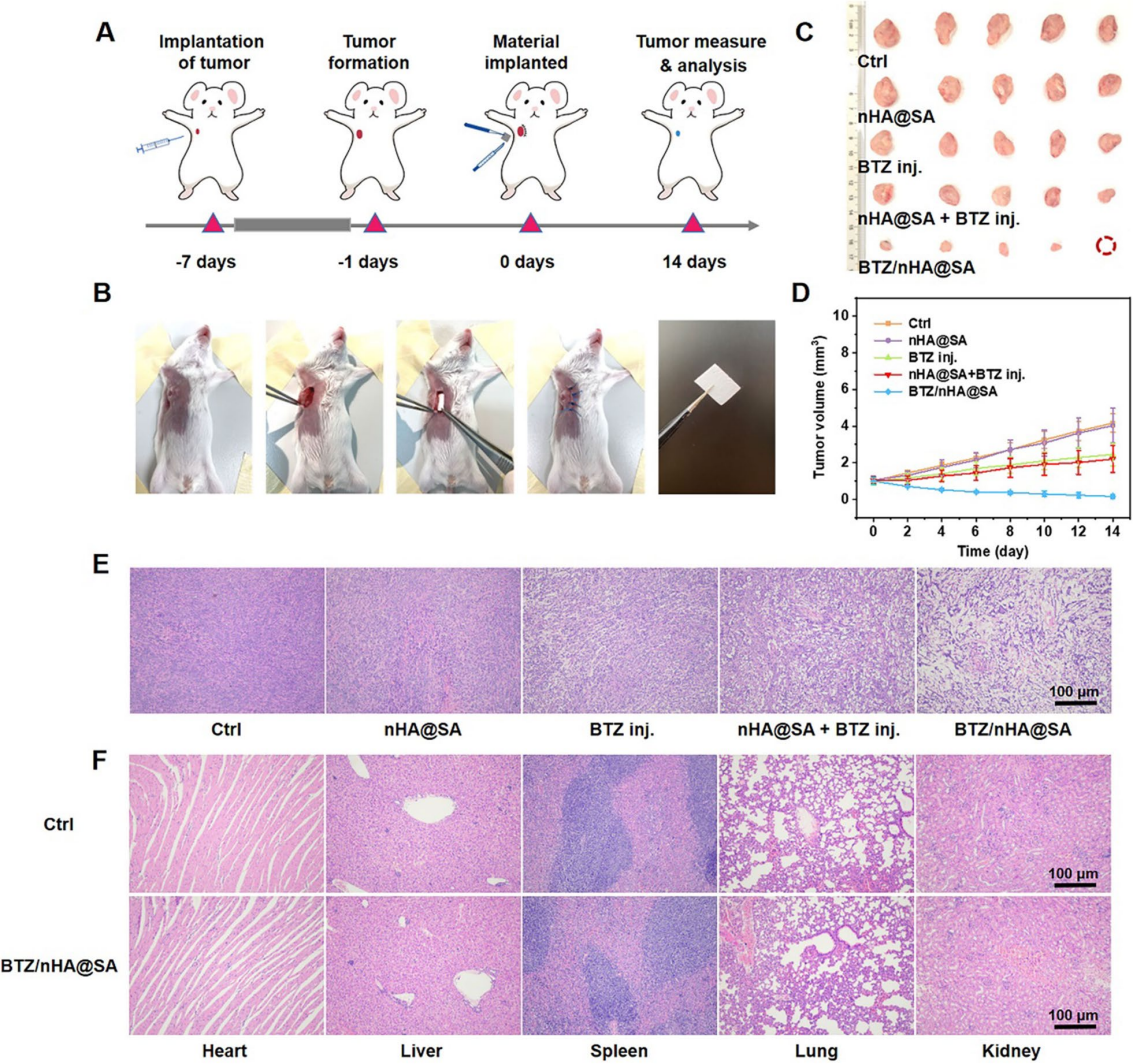
**A** Schematic diagram of anti-tumor experiment analysis in mice; **B** Flow chart of anti-tumor surgery in mice; **C** Pictures of the isolated tumors collected at day 14 after different treatments; **D** Tumor growth curves of mice subjected to different treatments; **E** H&E staining of tumor tissue slices; **F** H&E staining of major organs (heart, liver, spleen, lung and kidney) slices

In vivo, the anti-tumor capacity of BTZ/nHA@SA was investigated using a mouse breast cancer tumorigenesis test. BTZ/nHA@SA was fabricated as a thin film and implanted into the tumor site (Fig. 4A, B). During 14-day experimental period, the body weights of all five groups maintained a steady increase (Additional file 1: Fig. S5A), indicating that BTZ/nHA@SA scaffold had good biocompatibility. The tumors were removed after 14 days. The final tumor weights and sizes for all mice were shown in (Additional file 1: Fig. S5B, Fig. 4C). The most significant tumor volume reduction was found in the BTZ/nHA@SA group, followed by intra-tumoral injection of BTZ, and nHA@SA combined with BTZ intra-tumoral injection (Fig. 4D). Although the amount of BTZ in the intra-tumoural injection was the same as the total amount of drug loaded in the scaffold, the anti-tumor effect displayed a significant difference (*p* < 0.05), indicating that the intra-tumoral injection of BTZ was less effective than that of the sustained release from BTZ/nHA@SA scaffold and some injected BTZ may be leaked into systemic circulation metabolism. In contrast, BTZ/nHA@SA scaffold can maintain effective local drug concentrations for a longer time during the sustained release process, thus improving drug bioavailability.

HE staining results revealed that tumor cells in BTZ/nHA@SA group were severely damaged, with concentrated nuclei, vacuoles, and altered cell shape, indicating apoptosis of a large number of tumor cells (Fig. 4E). To evaluate the tissue toxicity of the BTZ/nHA@SA scaffold, we further examined histological sections of major organs, including heart, liver, spleen, lung, and kidney. There was no evidence of tissue inflammation or apoptosis in the BTZ/nHA@SA scaffold (Fig. 4F). White blood cell and red blood cell counts were both within the normal range, and no statistical difference between the BTZ/nHA@SA and control groups (*p* > 0.05), indicating no inflammatory foreign body reactions (Additional file 1: Fig. S5C–D). These results demonstrated that BTZ/nHA@SA scaffold can significantly inhibit tumor growth in vivo and had very good biological safety.

### In vivo osteogenic effects of BTZ/nHA@SA

**Fig. 5:**
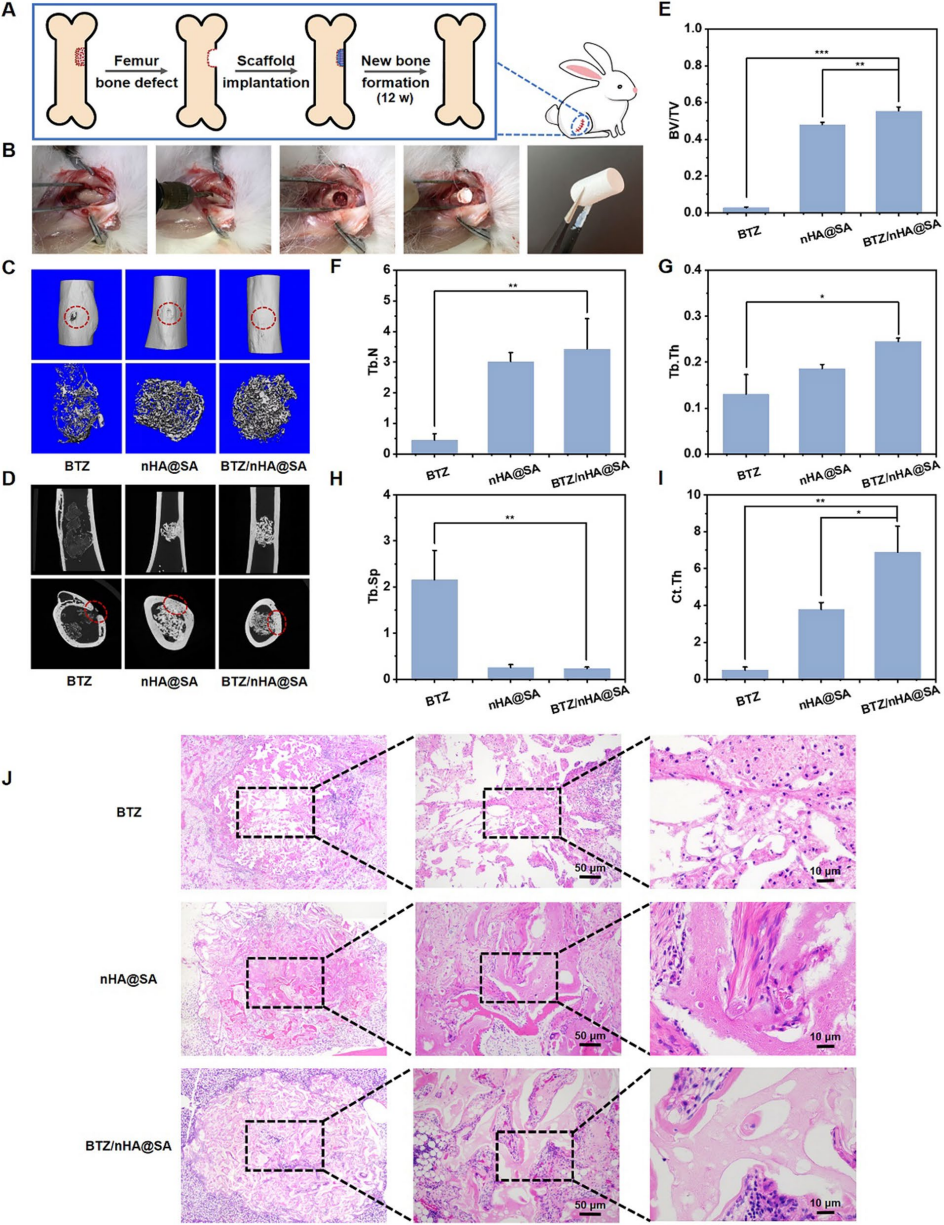
**A** Schematic diagram of rabbit bone regeneration experiment; **B** Flow chart of scaffold implantation in rabbit bone defect; **C** Micro-CT reconstruction images of femoral defect; **D** Coronal and transverse micro-CT reconstruction images of new bone at defect site; **E** Volume fraction of new bone in the area of the bone defect (BV/TV); **F–I** Trabecular number (Tb.N), trabecular thickness (Tb.Th), trabecular separation (Tb.Sp) and cortical bone thickness (Ct.Th) of new bone in the defect area by micro-CT quantitative analysis; **J** H&E staining of new bone tissue slices. *p < 0.05, **p < 0.01, ***p < 0.001

A rabbit femoral bone defect model was constructed to evaluate the in vivo osteogenic capacity of BTZ/nHA@SA scaffold. A cylindrical scaffold (6 mm in diameter and 9 mm in depth) was inserted into the bone defect area of the femoral bone marrow cavity (Fig. 5A, B). Samples were collected after 12 weeks for analysis. As can be seen from Additional file 1: Fig. S6A, complete healing of the bone defect was observed from the group treated with BTZ/nHA@SA scaffold in comparison with those of the control and nHA@SA groups.

The micro-CT analysis indicated that 12 weeks post-surgery, there was a significant new bone formation in the defect area for the BTZ/nHA@SA scaffold group (Fig. 5C, D). The area of a bone defect covered by new bone tissue in the BTZ/nHA@SA scaffold group was significantly higher (*p* < 0.05) than that of BTZ or nHA@SA group. The mineralized tissue regions were larger than the original cylindrical defect area, as exhibited in Fig. 5C. This phenomenon suggested that nHA@SA scaffold was beneficial for guiding bone cells growth from the periphery to the interior of the scaffold, promoting the uniform mineralization of bone tissue throughout the scaffold. The release of BTZ and Ca^2+^ from BTZ/nHA@SA scaffold may be able to guide the migration of osteoblast cells for bone repair.

From a cross-section of the reconstructed 3D bone defect, it can be seen that the cortical defect in the BTZ group was not entirely healed, while a dense and continuous bone cortex regeneration was observed in BTZ/nHA@SA and nHA@SA groups (Fig. 5D). This proved that both can induce the formation of well-organized bone cortex.

A quantitative analysis of the 3D reconstruction of bone trabeculae in the medullary cavity and surrounding cortical bone defect area suggested that the BV/TV (bone trabeculae volume fraction) in BTZ/nHA@SA group was significantly higher than that in nHA@SA and BTZ groups (*p* < 0.05; Fig. 5E). The trabecular number (Tb.N), trabecular thickness (Tb.Th) and cortical bone thickness (Ct.Th) showed a similar consistent trend too (Fig. 5F–H). 12 weeks after the operation, the trabecular separation (Tb.Sp) in BTZ/nHA@SA group was significantly lower than those in the other two groups (*p* < 0.05; Fig. 5I).

H&E sections of the bone tissues confirmed the histological formation and maturation of new bone after scaffold implantation. No significant inflammatory response was observed in all the groups (*p* > 0.05; Fig. 5J). The histological sections revealed that the defect area in BTZ group was mainly filled with fibrous tissue in the medullary cavity, and the number of bone trabeculae was still low after 12 weeks. In contrast, the defect area in the other two groups was mainly composed of well-structured mineralized new bone, which contained abundant osteocytes, and the new bone was wrapped with a collagen-rich extracellular matrix.

Quantitative analysis of trabecular bone counting presented that the density of the newborn trabecular bone in BTZ/nHA@SA group was much higher than that in the other two groups (Additional file 1: Fig. S6B). The bone induction performance of BTZ/nHA@SA was much higher than that of nHA@SA or BTZ group, and the newly formed bone structure was highly organized. Particularly, the quantitative micro-CT analysis and trabecular bone counting showed that BTZ/nHA@SA scaffold was highly effective to promote bone repair than any other group, indicating that the synergistic effect of BTZ-loaded nHA@SA scaffold had a “1 + 1 > 2” effect from a controlled combination.

## Discussion

In this work, a novel bifunctional BTZ/nHA@SA scaffold based on CDT local drug delivery was developed for bone tumor treatment and synchronous repair of neoplastic bone defects.

In addition to the anti-tumor effect, BTZ, a proteasome inhibitor used in chemotherapy, can also promote osteogenic responses under a relatively low concentration [27, 50]. We viewed that BTZ could be a potential drug for neoplastic bone defects, as BTZ was found to inhibit osteoclast differentiation through ubiquitin-mediated SMURF and NF-κB pathway, thereby reducing OVX-induced osteoporosis in mice [28, 51, 52]. At the same time, BTZ can mediate bone metabolism by increasing osteogenic activity and preventing mechanical unloading resulting in bone loss in mice [30]. However, few studies explored the application of BTZ in localized drug delivery. Previous studies have focused on the slow-release performance of BTZ but hardly noticed the biological interaction. As a proteasome inhibitor, BTZ can suppress CAV-1, a crucial protein regulating calcium influx [53]. It is found that reducing the expression of CAV-1 helped maintain the low differentiated state of MSCs and inhibit osteoclast, thereby inversely enhancing osteogenic activity [54, 55]. However, in addition to cell differentiation, calcium metabolism is also an indispensable part of bone repair. Upon CAV-1 inhibition, ET-1 mediates a decreased intracellular calcium concentration, which suggests that the BTZ-induced osteogenic effect may be limited by Ca^2+^ metabolism rather than cell differentiation [56, 57]. Therefore, we speculated that a sustained Ca^2+^ supply could improve BTZ-induced osteogenic activity by alleviating the calcium metabolism bottleneck resulting from CAV-1 inhibition. As a sequence, BTZ/nHA@SA scaffold demonstrated an excellent osteogenic performance beyond our expectations. With sustained Ca^2+^ releasing from nHA, internal–external communication of Ca^2+^ across cellular membrane continuously increased, thus promoting Ca^2+^ influx and improving Ca^2+^ metabolism. However, our hypothesis and the mechanism of the bifunctional BTZ/nHA@SA scaffold still need further research. A study presented that blocking the Ca^2+^ channel can reduce BTZ resistance through the NF-κB pathway via TG2, suggesting that Ca^2+^ supply may affect the anti-tumor effect of BTZ [58].

SA is widely recognized as having good biocompatibility and non-antigenicity without causing an immune-inflammatory response [39, 59], and its degradation rate could be controlled [60]. An ideal scaffold should provide sufficient strength before bone regeneration is fully completed. The incorporation of nHA nanoparticles enhanced the mechanical strength of the scaffold, but high stability can lead to the dilemma of secondary surgery for its removal. The BTZ/nHA@SA scaffold was fully degraded after 12 weeks, and no residue of SA was found from thorough inspections of the bone tissue sections. Considering the long osteogenesis period (12 weeks), we speculated that SA might be metabolized via the endocytosis of macrophages from bone marrow.

In this study, nHA was innovatively used as the SA crosslinking initiator and inorganic nanoparticle filler. GDL mediated the slow release of Ca^2+^ from nHA to promote the internal crosslinking of SA, thus effectively improving the mechanical strength of SA gel and providing cell adhesion sites and bone mineralization units [61]. This synthesis process required no additional calcium supply as an additional crosslinking initiator was introduced. Nanoparticles themselves supplied Ca^2+^ rather than using a liquid crosslinking initiator eliminating the possibility of insufficient or inhomogeneous mixing, and solving the problem of uneven reaction. As a result, the crosslinking of SA was more uniform and the scaffold had a more consistent structure and porosity. The prolonged Ca^2+^ release and the slow neutralization of H^+^ were thus capable of coordinating with the rate of bone regeneration.

In clinical application scenarios, scaffold materials for repairing tumorous bone defects require the property of synchronous tumor killing and bone generation. Ideally, in addition to sequentially and controllably releasing killing substances, a scaffold should provide an adequate pore size allowing cell colonization, structure stability under tumor acidic environment, pH response for favoring osteogenic induction, self-supply of Ca^2+^, and spontaneous degradation during the process of bone formation. This novel BTZ/nHA@SA scaffold has been developed to meet the above requirements by skillful utilization of BTZ with dual functions of tumor cell killing and osteogenic ability in BTZ concentration- and Ca^2+^-dependent manners. This material has significant advantages over traditional surgical performances, requiring no further follow-up treatments, including radiotherapy and systemic chemotherapy. In comparison to photothermal or photodynamic treatment, it eliminates the side effect of photothermal treatment where the high temperature could also kill osteoblasts. In summary, BTZ/nHA@SA offers an innovative therapeutic strategy for treating neoplastic bone defects.

## Conclusion

We designed a novel therapeutic nanocomposite scaffold with dual functions of simultaneous tumor inhibition and bone defect regeneration to meet a clinical challenge. Given the difficulty of repairing tumor bone defect sites and essential CDT treatment strategy, we specifically developed a BTZ-loaded nHA@SA bionic bone composite scaffold with the advantages of local tumor-killing drug delivery, Ca^2+^ supply tumor inhibition, and spontaneous tissue engineering repair.

BTZ/nHA@SA scaffold displays desirable microstructures, biodegradability, biocompatibility, and biological activity, and has achieved excellent tumor-killing and osteogenic activity. To meet clinical challenges, this work addresses neoplastic bone defects through the development of novel multifunctional scaffolds with both therapeutic and regenerative capabilities.

## Supplementary Information

**Additional file 1.** Additional Table S1, Figs. S1–S6.

## Data Availability

The raw date required to reproduce these findings are available to download from the manuscript. The processed data required to reproduce these findings are available to download from the manuscript.
