# Supplementary material for: A bifunctional bortezomib-loaded porous nano-hydroxyapatite/alginate scaffold for simultaneous tumor inhibition and bone regeneration
Source: J Nanobiotechnology. 2023 Jun 1;21:174. doi: 10.1186/s12951-023-01940-0 (PMC10236870; doi:10.1186/s12951-023-01940-0)
Supplement: Supplementary file 1 — Additional file 1. Additional Table S1, Figs. S1–S6. [file 12951_2023_1940_MOESM1_ESM.docx]

Additional Information

A bifunctional bortezomib-loaded nano-hydroxyapatite / alginate scaffold for simultaneous tumor inhibition and bone regeneration

*Jiafei Chen^1,#^, Junru Wen^1,#^, Yike Fu^2, 3,#^, Xiang Li^2, 3,*^, Jie Huang,^4^ Xiaoxu Guan^1,*^, Yi Zhou^1,*^*

^1^The Affiliated Hospital of Stomatology, School of Stomatology, Zhejiang University of Medicine, and Key Laboratory of Oral Biomedical Research of Zhejiang Province, Hangzhou, Zhejiang, 310006, China

^2^State Key Laboratory of Silicon Materials, School of Materials Science and Engineering, Zhejiang University, Hangzhou, P.R. China

^3^ZJU-Hangzhou Global Scientific and Technological Innovation Center, Zhejiang University, Hangzhou, 311215, China

^4^ Department of Mechanical Engineering, University College London, London WC1E 7JE, UK

**^#^**Authors with equal contribution: Jiafei Chen, Junru Wen and Yike Fu

*Corresponding Author: Xiang Li, Xiaoxu Guan and Yi Zhou

**Table S1.**

**Gene** **Primers** **Length**

133 bp

259 bp

161 bp

116 bp

123 bp

5’- CCTCGTCCCGTAGACAAAATG -3’

3 - TGAGGTCAATGAAGGGGTCGT -5’

5’- GATAACGAGATGCCACCAGAGG -3’

3’- GTTCAGTGCGGTTCCAGACATAG -5’

5’- CAAAGAAGCCATACGCTGACCT -3’

3’- AGGAAATGAGTGAGGGAAGGGT -5’

5’- ACACTGCCACCTCTGACTTCT -3’

3’- GGATGAAATGCTTGGGAACTGC -5’

5’- TCCCTACTCAGCCGTCTGTG -3’

3’- CCTCGCTTCCGTACTCGAAC -5’

GADPH

ALP

SP7

RUNX2

COL1

**
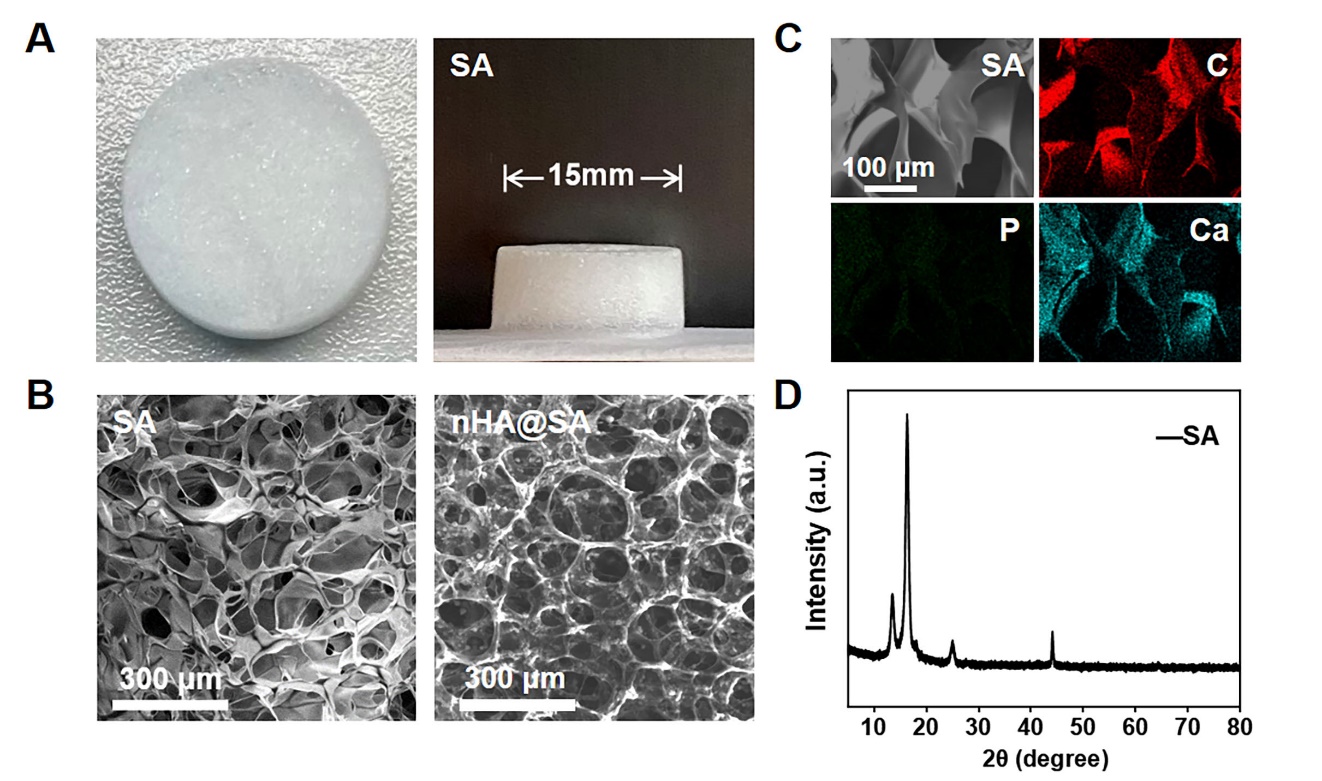
**

**Fig. S1.** (A) Photograph of SA scaffold; (B) FESEM images of SA scaffold and nHA@SA scaffold; (C) [EDS](https://www.sciencedirect.com/topics/engineering/energy-dispersive-spectrometer) elemental mapping analysis shows the uniform element distribution of Ca and C; (D) XRD pattern of SA.

**
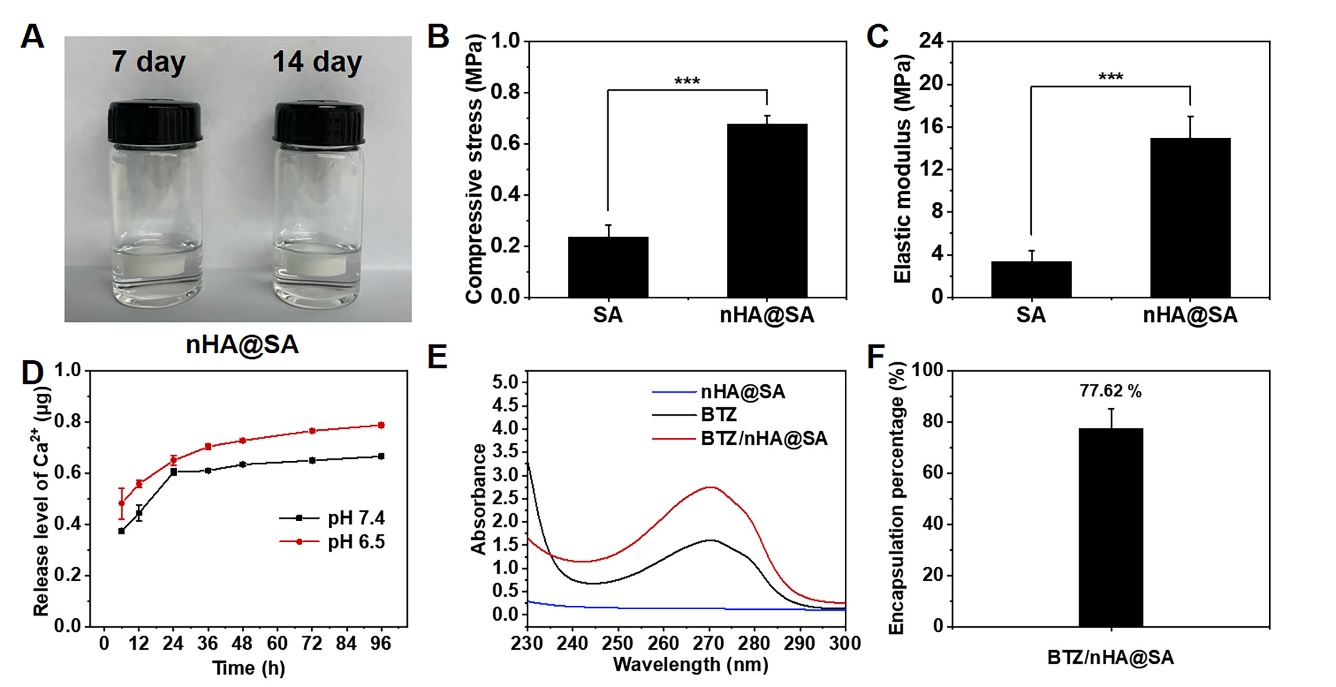
**

**Fig. S2.** (A) Photograph of nHA@SA scaffold during degradation; (B) Compressive strength of SA scaffold and nHA@SA scaffold; (C) Elastic modulus of SA scaffold and nHA@SA scaffold; (D) Release level of Ca^2+^ from nHA@SA scaffold at pH 6.5 and 7.4; (E) UV-vis absorption curves of nHA@SA scaffold, BTZ and BTZ/nHA@SA; (F) Encapsulation rate of BTZ in BTZ/nHA@SA scaffold. ****p* < 0.001.

**
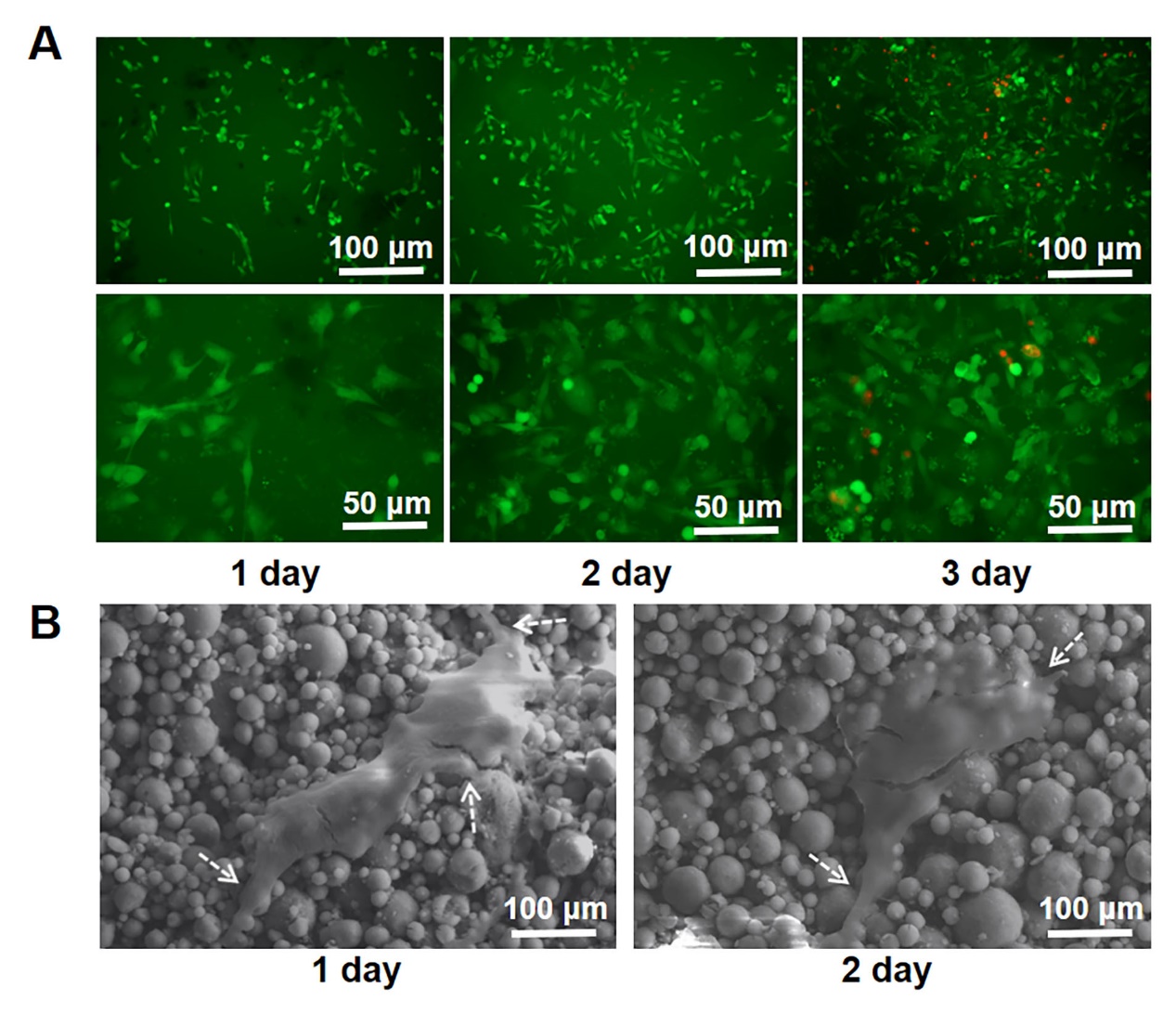
**

**Fig. S3.** (A) Fluoresence images of MC3T3 cells cultured with BTZ/nHA@SA scaffold stained with calcein AM (green, live cells) and PI (red, dead cells) at day 1, 2 and 3; (B) SEM micrographs of the attachment of MC3T3 cells on BTZ/nHA@SA scaffold at day 1 and 2 after co-culture.

**
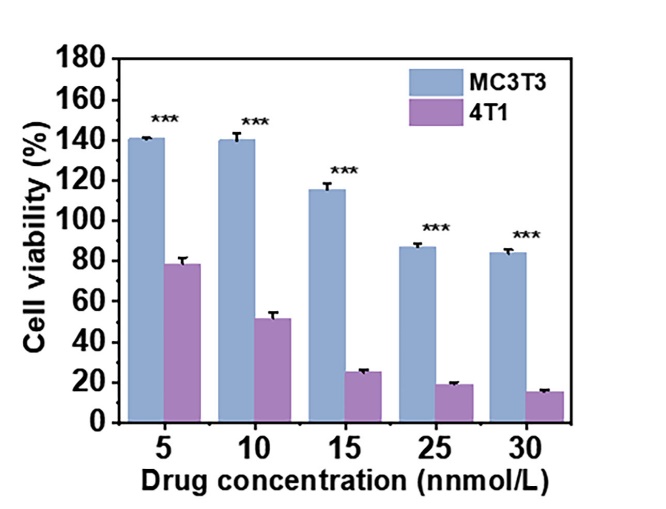
**

**Fig. S4.** The effects of different BTZ concentration on the viability of MC3T3 cells and 4T1 cells. ****p* < 0.001.

**
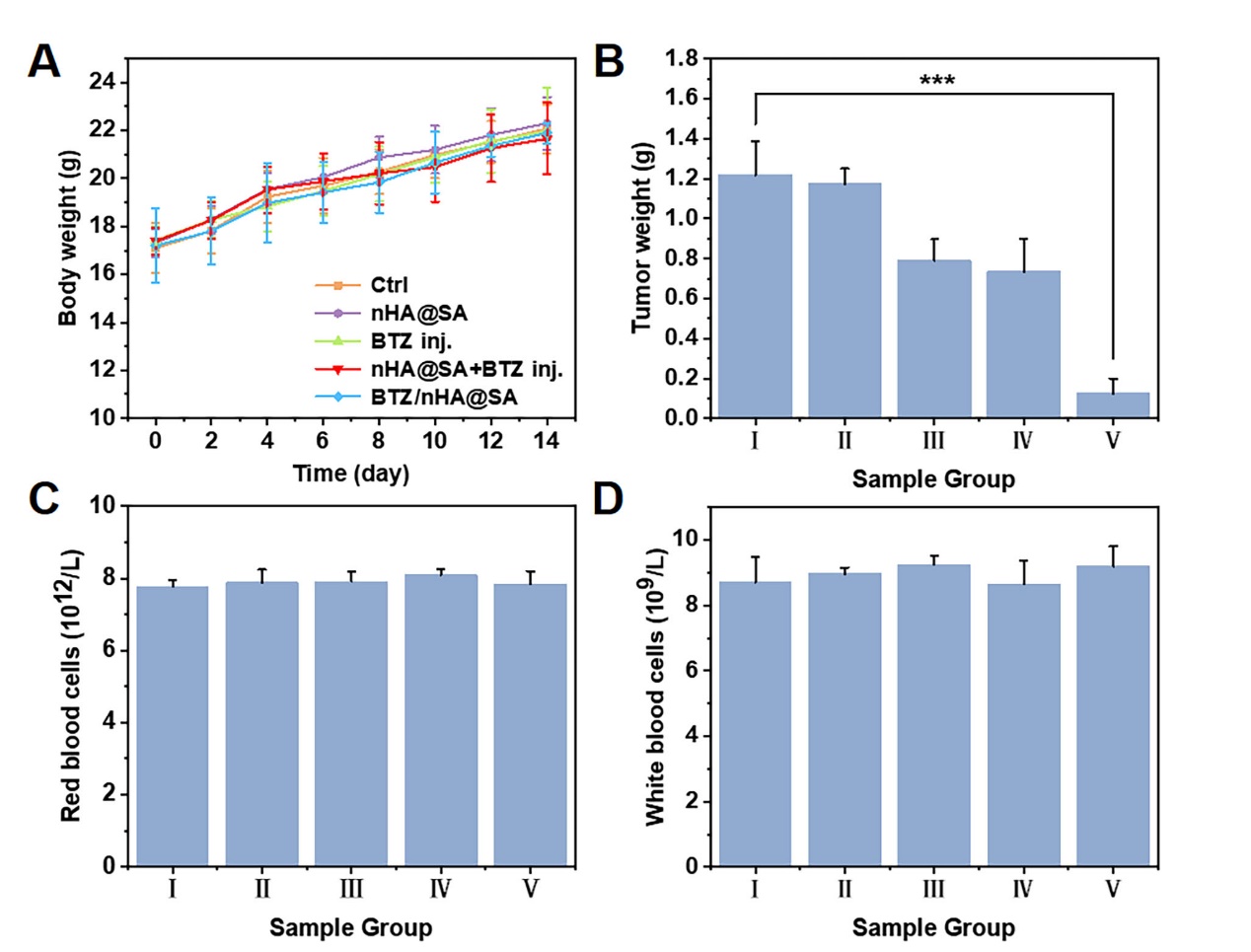
**

**Fig. S5.** (A) Body weight changes of mice subjected to different treatments; (B) Tumor weight (C) Red blood cell and (D) White blood cell levels of mice after different treatments for 14 days ( I: Ctrl, sham operation; II: implantation of nHA@SA scaffolds; III: injection of BTZ; IV: BTZ drug injection＋nHA@SA scaffold implantation; V: BTZ/nHA@SA scaffold implantation). Hematological data are within the reference range. ****p* < 0.001.

**
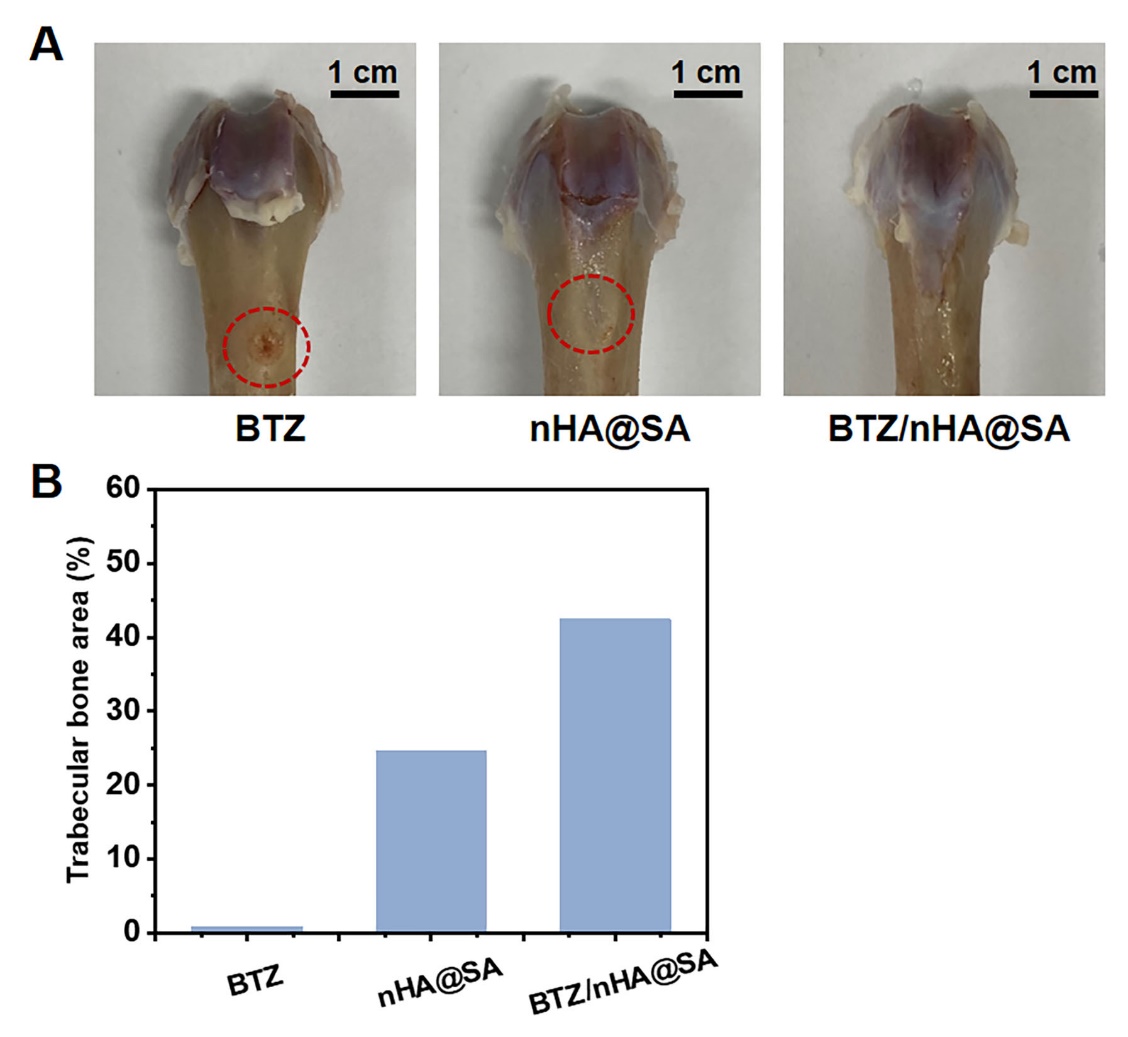
**

**Fig. S6.** (A) Photograph of rabbit bone defect 12 weeks after surgery; (B) A comparison of new bone formation of rabbit after different treatments for 12 weeks.
